# Concept of a fast breeder reactor to transmute MAs and LLFPs

**DOI:** 10.1038/s41598-021-01986-w

**Published:** 2021-11-17

**Authors:** Toshio Wakabayashi

**Affiliations:** Graduate School of Engineering, Tohoku University, 6–6–11 Aoba, Aramaki, Aoba-ku, Sendai, Miyagi 980–8579 Japan

**Keywords:** Energy science and technology, Physics

## Abstract

The long-term issues of nuclear power systems are the effective use of uranium resources and the reduction of radioactive waste. Important radioactive wastes are minor actinides (MAs: ^237^Np, ^241^Am, ^243^Am, etc.) and long-lived fission products (LLFPs: ^129^I, ^99^Tc, ^79^Se, etc.). The purpose of this study was to show a concept that can simultaneously achieve the breeding of fissile materials and the transmutation of MAs and LLFPs in one fast reactor. Transmutation was carried out by loading innovative Duplex-type MA fuel in the core region and LLFP-containing moderator in the first layer of the radial blanket. Breeding was achieved in the core and axial blanket. As a result, it was clarified that in this fast breeder reactor, a breeding ratio of approximately 1.1 was obtained, and MAs and LLFPs achieved a support ratio of 1 or more. The transmutation rate was 10.3%/y for ^237^Np, 14.1%/y for ^241^Am, 9.9%/y for ^243^Am, 1.6%/y for ^129^I, 0.75%/y for ^99^Tc, and 4%/y for ^79^Se. By simultaneously breeding fissile materials and transmuting MAs and LLFPs in one fast reactor, it will be possible to solve the long-term issues of the nuclear power reactor system, such as securing nuclear fuel resources and reducing radioactive waste.

## Introduction

Important issues for nuclear power generation are the effective use of uranium resources and the reduction of radioactive waste while ensuring safety. For the effective use of uranium resources, the fast breeder reactor (FBR) can convert ^238^U to fissile material, such as ^239^Pu, with a breeding ratio of 1 or more.

To reduce radioactive waste, the amounts of minor actinides (MAs: ^237^Np, ^241^Am, ^243^Am, etc.) and long half-lived fission products (LLFPs: ^99^Tc, ^129^I, ^79^Se, etc.) must be reduced. In back-end research, MAs are the major elements of the potential toxicity of radioactive waste. By recovering and transmuting minor actinide nuclides, it is thought that the potential toxicity after 1000 years can be reduced to 1/100. In addition, the removal of MAs is said to be effective in reducing the area of the disposal site. On the other hand, LLFPs are considered important in terms of the radiation safety performance (future exposure dose to the public) of the disposal site.

Many studies have been conducted on the transmutations of MAs and LLFPs in fast reactors^1–31^. Regarding MAs, if an average of approximately 5% MAs is added to the fuel region, a transmutation rate of 10%/y or more is achieved without significantly affecting the core characteristics^7,13^.

Regarding LLFPs, a transmutation study has recently been conducted on 6 important nuclides (^79^Se, ^93^Zr, ^99^Tc, ^107^Pd, ^129^I, and ^135^Cs) in terms of reducing environmental influence. As a result, it was shown that the fast reactor can be used to transmute each of these 6 nuclides with a support ratio exceeding 1 using the YD_2_ moderator^28^. A significant reduction in the effective half-life was obtained by analysis. In this case, the six nuclides were used as elements without isotope separation. In addition, a method that can transmute six nuclides at the same time with a support ratio of 1 or more in one fast reactor was clarified^29^. In this case, ^135^Cs and ^93^Zr, both with small neutron absorption cross sections, were placed in the radial blanket region, and ^129^I and ^99^Tc, both with large neutron absorption cross sections, were placed in the shield region and axial blanket away from the fuel region. Therefore, the transmutation rate of all nuclides was less than 0.5%/y. On the other hand, a study of a method designed to achieve a high transmutation rate (approximately 8%/y) for four nuclides (^79^Se, ^99^Tc, ^107^Pd, ^129^I) was conducted^30^. Based on these studies, a system that further improves the transmutation efficiency of the six nuclides was explored. A fast reactor LLFP transmutation system that achieves a support ratio of 1 or more for the entire system was constructed by combining three fast reactors, in addition to using one reactor^31^. From these studies, a substantial amount of information about the LLFP transmutation system was obtained.

Among these LLFP nuclides, ^129^I presents a long-term radioactivity problem in geological disposal as a long-lived nuclide that is soluble and less absorbed by underground materials^32^. ^99^Tc is the main radioisotope of vitrified radioactive waste, and its potential toxicity is a problem. ^79^Se has been a determinant of radiation exposure for 10^4^–10^5^ years. Reducing these three nuclides could reduce the uncertainty of geological disposal. Therefore, these three nuclides were selected as LLFPs for transmutation in this study.

If breeding of fissile materials and transmutation of MAs (^237^Np, ^241^Am, ^243^Am) and LLFPs (^129^I, ^99^Tc, ^79^Se) can be performed simultaneously in one fast reactor, the long-term issues of securing nuclear fuel resources for nuclear power systems and reducing radioactive waste would be solved. In addition, solutions to these issues would lead to a better understanding of nuclear power generation among the general public. The purpose of this study was to show a system that simultaneously achieves the breeding of fissile materials and the transmutation of MAs and LLFPs in one fast breeder reactor.

Here, the support ratio (SR) was defined as the ratio of the amount of each nuclide transmuted by the fast breeder reactor to the amount of each nuclide (MAs and LLFPs) produced by the fast breeder reactor^28,30,31^.

## Results and discussions

Studies on MA transmutation in fast reactors have investigated homogeneous loading, in which MAs are homogeneously added to the core fuels, and heterogeneous loading, in which assemblies of only MAs are loaded in the core in a dispersed manner. In the case of homogeneous loading, the effect on the power distribution in the core was small, but because of the strong radiation of MAs, large-scale shielded cells were required to prevent exposure during manufacturing. In the case of heterogeneous loading, the MA assemblies with large neutron absorption cross sections and the normal MOX fuel assemblies were mixed, so the difference in power among these assemblies was large, and the power distribution in the core was difficult to control.

**Figure 1:**
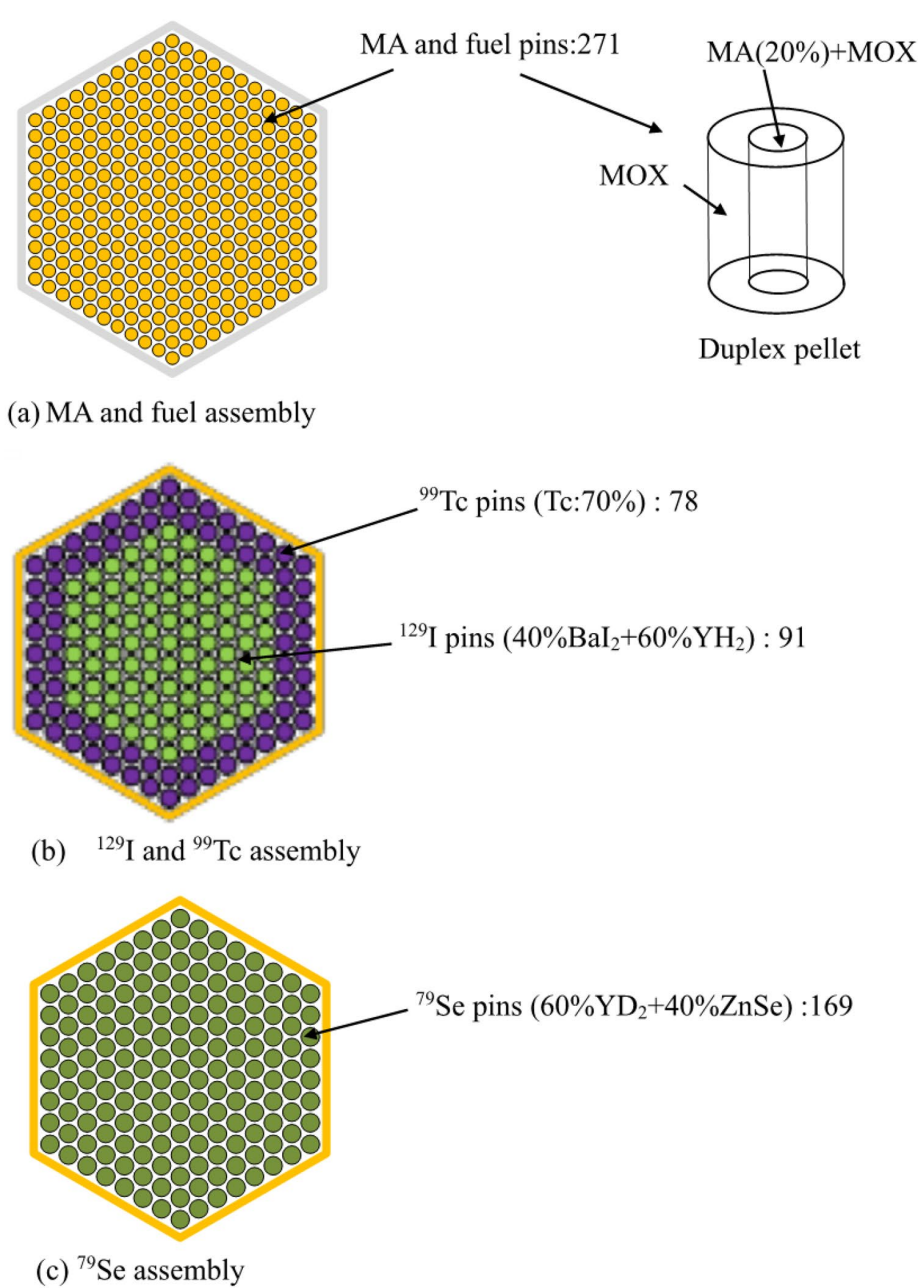
Configurations of MA fuel assembly, ^129^I and ^99^Tc assembly, and ^79^Se assembly. (**a**) Structure of the MA-containing MOX fuel assembly. The number of MA fuel pins in the MA-containing fuel assembly is 271. (**b**) Arrangement of the ^99^Tc pins and the ^129^I pins in the assembly. Eighty-six ^99^Tc and ^129^I assemblies are loaded in the first layer of the blanket region. (**c**) A total of 169 pins in the form of mixed ZnSe and YD_2_ are arranged in the assembly. Ten ^79^Se assemblies are placed in the first layer of the blanket region.

**Figure 2:**
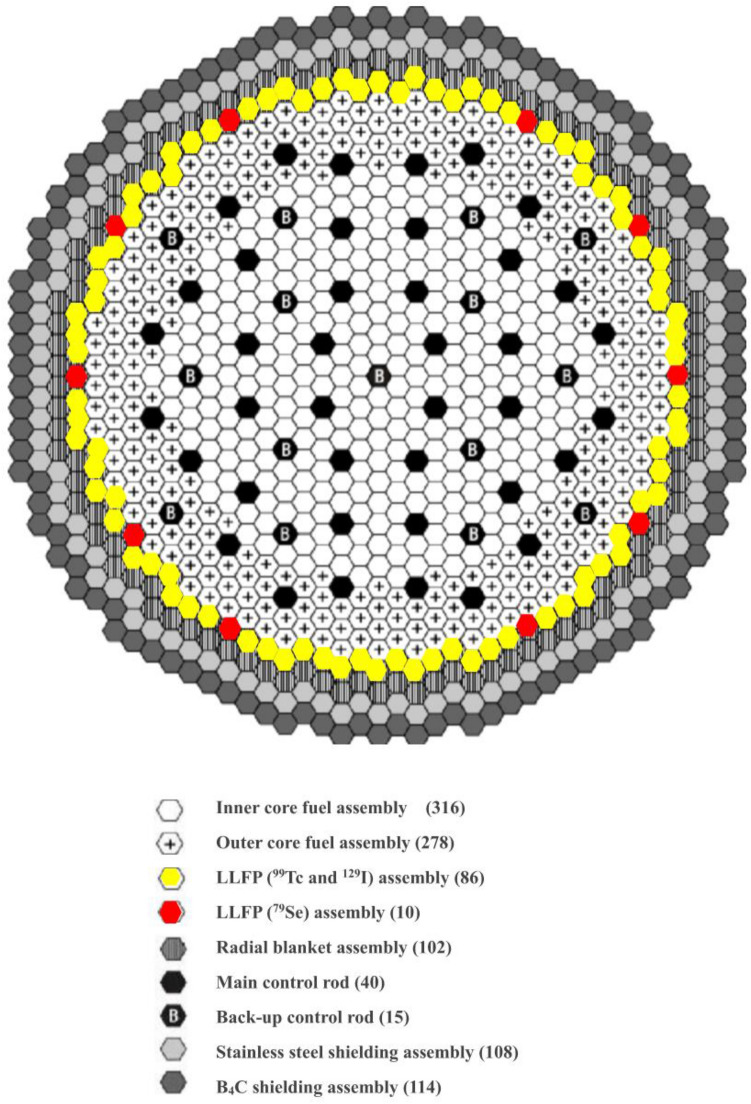
Core arrangement for MA and LLFP transmutation in a fast breeder reactor. The core has two homogeneous zones: inner and outer cores. MA fuel assemblies are loaded in the inner and outer cores. The LLFP assemblies are loaded in the first layer of the blanket region. The blanket fuel assemblies are loaded in the second layer of the blanket region.

Therefore, duplex-type MA fuel was proposed as an innovative alternative. The concept of MA fuel is to insert the MA pellet into the center of the hollow MOX pellet, as shown in Fig. 1a. This is called a Duplex pellet. Normal hollow MOX pellets can be manufactured in glove boxes that do not require shielding to prevent high radiation exposure. The central MA pellet is manufactured in a small shielding facility and inserted into the center of the hollow MOX pellet. In the case of MA homogeneous fuel, a large-scale shield cell is required because MAs are handled in all processes. On the other hand, duplex-type MA fuel is expected to simplify manufacturing equipment and reduce manufacturing costs. Figure 1a shows the structure of the MA-containing MOX fuel assembly. The number of MA fuel pins in the MA fuel assembly was 271. The MA content of the central MA pellet was set to 20 wt% so that the average of MOX and MA pellets was 5 wt%. MA-containing MOX fuel assemblies were loaded in the inner and outer cores. Figure 2 shows the arrangement of MA-containing fuel assemblies in the fast breeder reactor core. Regarding the fabrication of duplex pellets, studies were conducted on the effects of Gd_2_O_3_ placed in the center of oxide fuel pins in LWR^33,34^. In addition, by using Duplex pellets containing neutron absorbers in fast reactors, a new core concept has been proposed that did not achieve recriticality in the case of fuel melting in an accident^35^. From these studies, the innovative duplex-type MA fuel used in MA transmutation is considered sufficiently feasible.

Regarding the transmutation of LLFPs, LLFP assemblies in which moderators^36–40^ (YH_2_, YD_2_, etc.) were combined with LLFP nuclides improved the transmutation performance of LLFPs. When an LLFP assembly that combines LLFPs and the YH_2_ moderator is loaded in the blanket region, YH_2_ has a high moderating ability, so the transmutation rate increases. However, an increase in the amount of thermal neutrons would create a thermal spike by causing the power of the adjacent fuel assembly to increase. To prevent a thermal spike, in the case of the ^129^I transmutation, ^99^Tc metal pins were installed in the outermost two layers, and ^129^I pins containing a mixture of BaI_2_ and moderator YH_2_ were installed in the inner part of the assembly. As a result, the thermal neutrons emitted from the fuel regions are absorbed by ^99^Tc, which has a large neutron absorption cross section, so that the thermal spike of the adjacent fuel can be reduced. Figure 1b shows the arrangement of the ^99^Tc and ^129^I pins in the assembly. As shown in Fig. 2, 86 assemblies of ^99^Tc and ^129^I were loaded in the first layer of the blanket region.

For ^79^Se, the transmutation rate did not change substantially regardless of whether the moderator was YD_2_ or YH_2_; therefore, YD_2_ was used to address the issue of the thermal spike. As shown in Fig. 1c, 169 pins in the form of mixed ZnSe^41,42^ and YD_2_ were arranged in the assembly^30,31^. As shown in Fig. 2, ten ^79^Se assemblies were placed in the first layer of the blanket region. This is because the amount of ^79^Se produced in the fast breeder reactor is as small as 0.22 kg/y.

**Figure 3:**
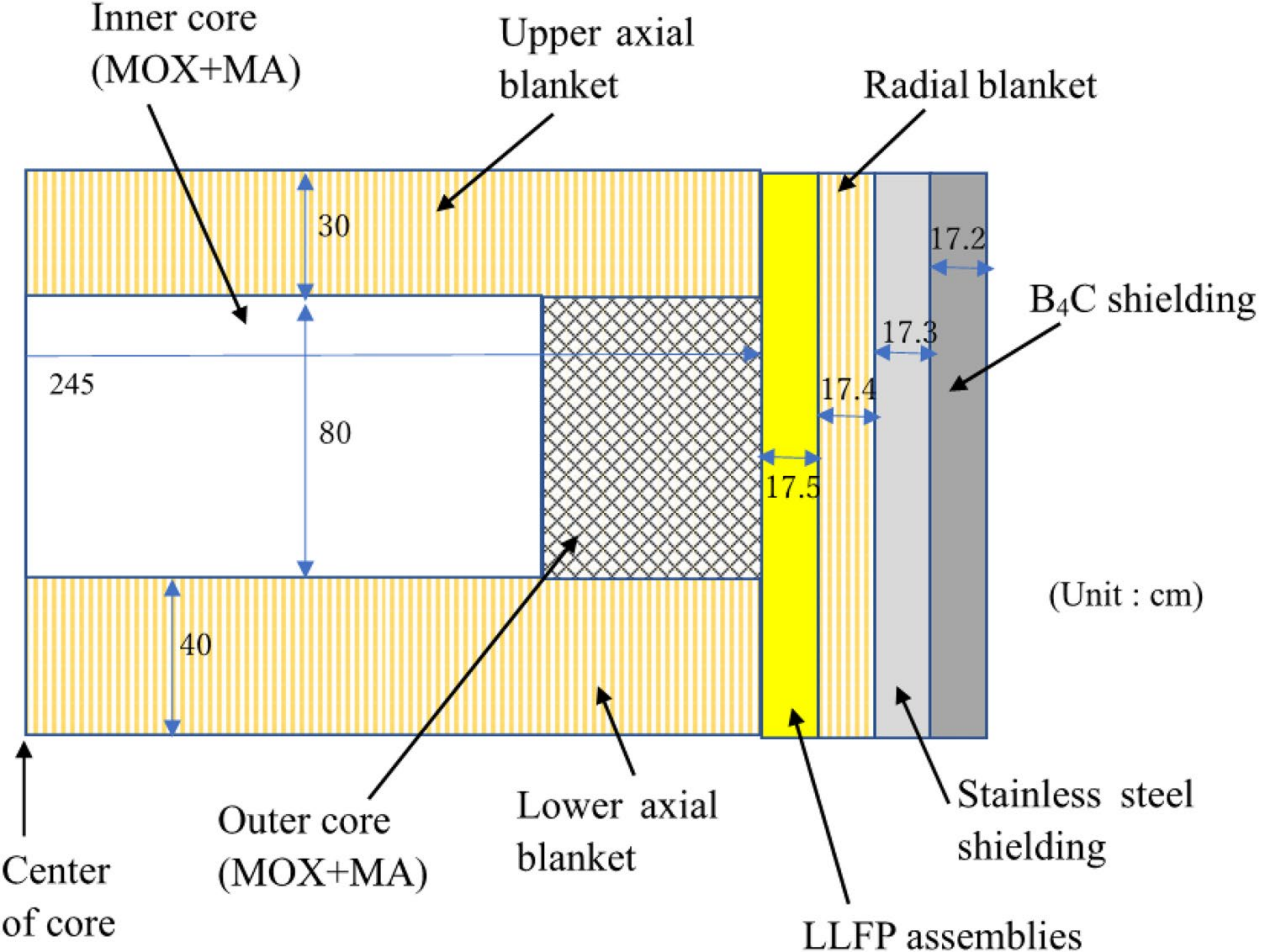
Axial core arrangement for MA and LLFP transmutation in a fast breeder reactor. The height of the core is 80 cm. The thicknesses of the upper and lower axial blankets are 30 cm and 40 cm, respectively. The core equivalent diameter is 490 cm.

The breeding of fissile materials by the axis blanket is considered possible, as substantial breeding by the radial blanket cannot be expected because the LLFP assemblies are loaded in the first layer of the radial blanket. The thicknesses of the upper and lower axial blankets were 30 cm and 40 cm, respectively. Figure 3 shows a cross-sectional view of the core.

**Figure 4:**
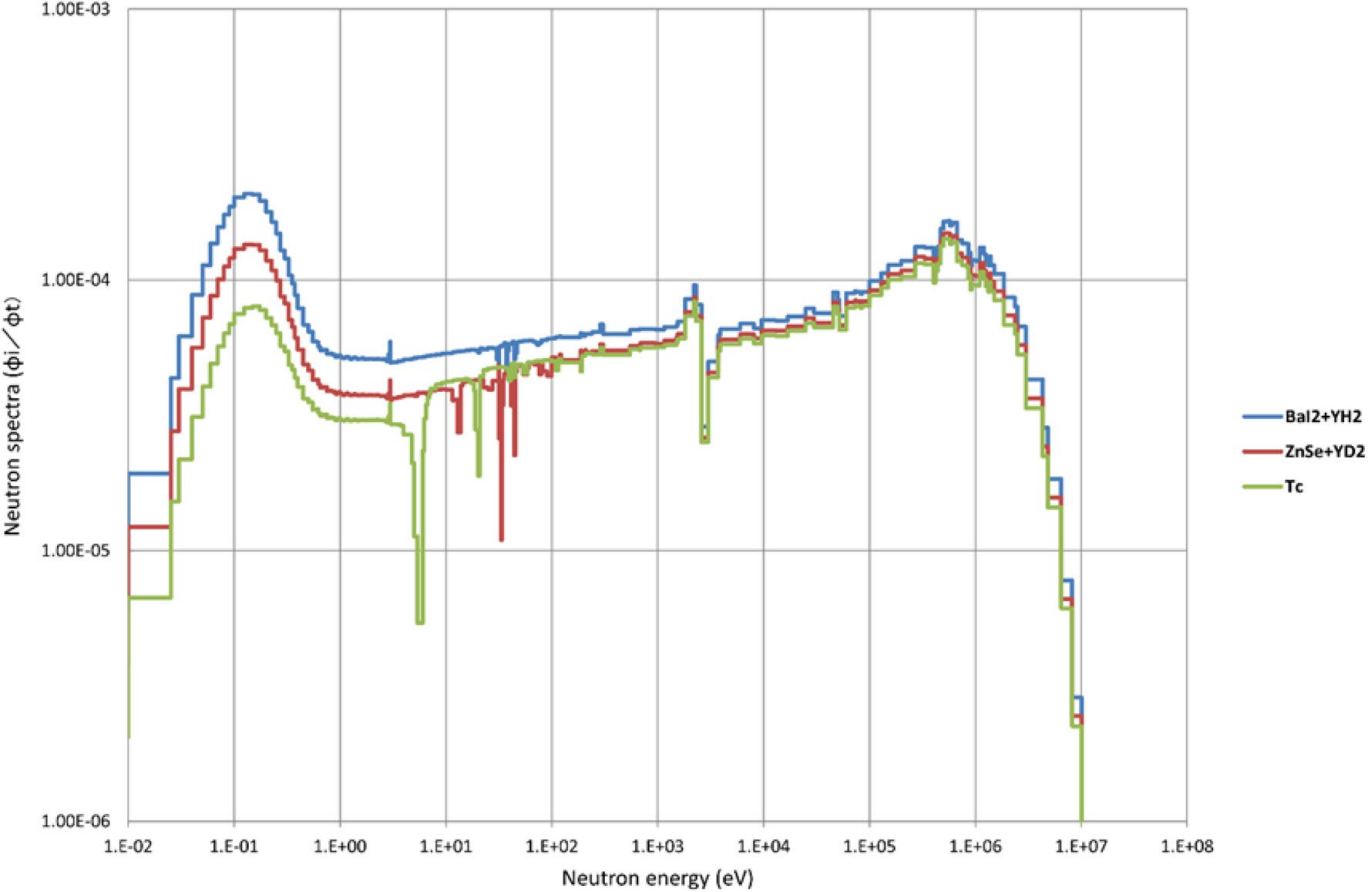
Neutron energy spectra in the LLFP assemblies. The neutron spectra show the cases of ^129I^ pin (BaI_2_ + YH_2_), ^99^Tc pin (Tc metal) and ^79^Se pin (ZnSe + YD_2_). The thermal neutron flux increases in the order of ^129^I pin, ^79^Se pin, and ^99^Tc pin.

Figure 4 shows the calculation results of the neutron energy spectrum of the LLFP assemblies. The neutron spectra show the cases of ^129^I pin (BaI_2_ + YH_2_), ^99^Tc pin (Tc metal) and ^79^Se pin (ZnSe + YD_2_). The thermal neutron flux increases in the order of ^129^I pin, ^79^Se pin, and ^99^Tc pin. This is because the thermal neutron flux increases in the order of using moderators with higher moderating capacity.

Table 1 shows the analysis results of MA transmutation. The composition of the loaded MA nuclides was based on the composition of fuel discharged from the fast breeder reactor, as shown in Table 2, and the composition of fuel discharged from a light water reactor was also studied as a reference. ^244^Cm was excluded from the loaded MA nuclides because it has a short half-life of 18.1 years, and Cm can be separated from MAs^43^. Since the MA composition of the fuel discharged from the fast breeder reactor changes due to the transition from Pu, Am nuclides increase. On the other hand, since the MA composition of fuel discharged from the LWR changes due to the transition from U, ^237^Np increases. In the case of the MA composition of fuel discharged from the fast breeder reactor, a support ratio of 1 or more can be achieved for each of the three MA nuclides. The transmutation rate was 10.3%/y for ^237^Np, 14.1%/y for ^241^Am, and 9.9%/y for ^243^Am. Since the transmutation characteristics are excellent, the fast breeder reactor system is considered capable of transmuting MAs flexibly according to the MA inventory. In the case of the MA composition of the fuel discharged from LWR, the support ratio was as large as 25.6 for ^237^Np, but it was just 1.0 for ^243^Am. Regarding the transmutation rate, ^237^Np and ^241^Am were greater than 10%/y, but ^243^Am was as small as 5.8%/y. When using MAs with the composition of the fuel discharged from LWR, efficient MA transmutation is considered achievable by mixing it with the MA composition of fuel discharged from the fast breeder reactor.

**Table 1:** Transmutation rate and support ratio of Mas.

Fuel composition	MA abundance of discharged FBR fuel			MA abundance of discharged LWR fuel		
Nuclide	^237^Np	^241^Am	^243^Am	^237^Np	^241^Am	^243^Am
Transmutation rate (%/y)	10.3	14.1	9.9	12.4	14.0	5.8
SR	4.6	6.3	4.4	25.6	4.6	1.0

**Table 2:** Isotope abundance and half-life of loaded MA nuclides.

	Fuel composition		Half-life (year)
	MA abundance of discharged FBR fuel (%)	MA abundance of discharged LWR fuel (%)	
^237^Np	11.3	50.5	2,144,000
^241^Am	51.3	37.0	433
^243^Am	37.4	12.5	7370

Table 3 shows the analysis results of LLFP transmutation. The SR is 1.13 for ^99^Tc, 1.24 for ^129^I, and 3.3 for ^79^Se, all exceeding 1. Regarding the transmutation rate, ^99^Tc was 0.79%/y, ^129^I was 1.24%/y, and ^79^Se was 3.3%/y. The transmutation rates of ^99^Tc and ^129^I were lower than the values obtained for 300 MWe class fast reactors (^99^Tc: 2.47%/y, ^129^I: 3.41%/y). This is because the leakage of neutron flux in the radial direction is reduced due to the increase in the core diameter. Since the number of loaded assemblies of ^79^Se was as small as 10, the transmutation rate of ^79^Se was not substantially affected by the size of the core.

**Table 3:** Transmutation rate and support ratio of LLFPs in a fast breeder reactor with MAs and LLFPs.

	^99^Tc	^129^I	^79^Se
Transmutation rate (%/y)	0.75	1.4	4.0
SR	1.13	1.24	3.3

Table 4 shows that compared with that of a normal large-scale fast breeder reactor (with a radial blanket), the breeding ratio of the fast breeder reactor loaded with MAs and LLFPs was slightly lower, but an approximately 1.1 breeding ratio could be obtained. This is because the contribution of the breeding ratio of the axial blanket in a large-scale fast breeder reactor is larger than that of the radial blanket, and the breeding ratio of the entire core does not decrease significantly even if the first layer of the radial blanket is replaced with the LLFP assemblies.

**Table 4:** Comparison of breeding ratios with and without MAs and LLFPs.

Core arrangement	FBR with MAs and LLFPs	FBR without MAs and LLFPs
Core	0.76	0.76
Axial blanket	0.32	0.32
Radial blanket	0.01	0.11
Total	1.09	1.19

The sodium void reactivity and the Doppler coefficients related to the safety of the fast breeder reactor loaded with MAs and LLFPs were analyzed. In Table 5, the sodium void reactivity and absolute value of the Doppler coefficient of the fast breeder reactor loaded with MAs and LLFPs are approximately 30% higher and approximately 40% smaller, respectively, than those of the normal large-scale fast breeder reactor. This is because the neutron spectrum of the core became harder due to the addition of MAs. However, these changes in the Doppler coefficient and sodium void reactivity are considered free from major safety problems.

**Table 5:** Characteristics of sodium void reactivity and Doppler coefficient in the core of the fast breeder reactor.

Core arrangement	FBR with MAs and LLFPs	FBR without MAs and LLFPs
Sodium void reactivity ($)	6.8	5.2
Doppler coefficient (Tdk/dT)	− 4.0E−3	− 6.6E−3

Regarding MAs, the difference in the transmutation rate between MA-containing duplex pellets and homogeneous MA pellets was analyzed. As shown in Table 6, no significant difference was found between the transmutation rates of MA-containing duplex pellets and homogeneous MA pellets, and MA-containing duplex pellets were effective. Since the energy spectrum of neutrons is hard in the core, the self-shielding effect of inserting MAs in the central region is considered small.

**Table 6:** Comparison between transmutation rates of duplex pellets and homogeneous pellets.

Pellet arrangement	Transmutation rate (%/y)	
	Duplex pellet with MAs in the center region (MAs:20%)	Homogeneous pellet with MAs (MAs:5%)
^237^Np	14.1	14.3
^241^Am	10.3	10.5

## Conclusions

From this study, the new concept of a fast breeder reactor system that can transmute MAs (^237^Np, ^241^Am, ^243^Am) and LLFPs (^129^I, ^99^Tc, ^79^Se) with a support ratio of 1 or more was constructed while breeding fissile materials in one fast breeder reactor. It was clarified that this fast breeder reactor achieved a breeding ratio of approximately 1.1 and MA and LLFP support ratios of 1 or more. The transmutation rate was 10.3%/y for ^237^Np, 14.1%/y for ^241^Am, 9.9%/y for ^243^Am, 1.6%/y for ^129^I, 0.75%/y for ^99^Tc, and 4%/y for ^79^Se.

Based on these studies, the following progress can be considered impactful for nuclear power development, effective use of uranium resources, and reduction of radioactive waste.

- By simultaneously breeding fissile materials and transmuting MAs and LLFPs in one fast reactor, it will be possible to solve the long-term issues of nuclear power systems, such as securing nuclear fuel resources and reducing radioactive waste. In addition, solving these issues would promote a better understanding of nuclear power systems among the general public.
- The ability to breed fissile materials and transmute MAs and LLFPs in one fast breeder reactor shows the high potential of the fast breeder reactor and will promote research and development of the fast breeder reactor.
- This new concept can contribute to the effective use of uranium resources and the reduction of radioactive waste without substantially changing the conventional nuclear fuel cycle system.

As future study subjects, since the transmutation rate of LLFP nuclides is low, methods to improve it must be studied. In addition, the establishment of manufacturing technology for MA-containing duplex pellets and cost evaluation can be mentioned.

## Method

### Core conditions

This study used a large sodium-cooled fast breeder reactor designed for the commercial stage. Table 7 shows the main specifications. The thermal power of the reactor was 3570 MWt, and the electric power was 1500 MWe. The core was a homogeneous two-region core, with 316 MA-containing fuel assemblies in the inner core, 278 MA-containing fuel assemblies in the outer core, and 55 control rods. The outside of the core was composed of 96 LLFP assemblies, 102 radial blanket assemblies, and 222 radial shielding assemblies. The height of the core was 80 cm. The core equivalent diameter was 490 cm. The Pu enrichment of the inner and outer cores were 20.7 W% and 23.3 wt%, respectively. The Pu enrichment of the outer core was higher than that of the inner core to achieve power flattening. Table 8 shows the specifications of the MA-containing fuel assembly and the LLFP assembly. There were 271 MA-containing fuel pins in the fuel assembly and 169 LLFP pins in the LLFP assembly. The isotopic compositions of MAs and LLFPs loaded in the fast breeder reactor shown in Tables 2 and 9 were based on the results of 80 GWd/t burnup simulation in the fast breeder reactor by MVP-BURN code. The MA composition of the discharged LWR fuel was based on the results of a 40 GWd/t burnup simulation of LWR UO_2_ fuel.

**Table 7:** Specification of large-scale fast breeder reactors for MA and LLFP transmutation.

Thermal power (MWt)	3570
Electric power (MWe)	1500
Core type	Homogeneous two-region core
Operation cycle length (months)	18
Number of refueling batches (Core/Blanket)	4/4
Core height (cm)	80
Thickness of axial blanket (cm) (Upper/Lower)	30/40
Number of core fuel assemblies (Inner/Outer/Total)	316/278/594
Number of LLFP assemblies	96 (^129^I and ^99^Tc assembly:86, ^79^Se assembly:10)
Number of radial blanket assemblies	102
Pu enrichment (wt%) (inner/outer core)	20.7/23.3
Number of control rods (Main/backup)	40/15
Number of radial shielding assemblies	108/114
Volume ratio of core (Fuel/Structure/Coolant)	44.1/24.2/31.7

**Table 8:** Specifications of MA-containing fuel assembly and LLFP assembly.

	MA-containing fuel assembly	LLFP assembly
Pin diameter (mm)	8.8	11.5
Thickness of pin (mm)	0.52	0.5
Pellet diameter (mm)	7.6	10.3
Number of pins in the assembly	271	169

**Table 9:** Isotope abundance and half-life of loaded LLFP nuclides.

Isotopes of loaded LLFP elements	Abundance (%)	Half-life of LLFPs (year)
^76^Se	0.027	
^77^Se	2.786	
^78^Se	5.587	
^79^Se	13.32	295,000
^80^Se	22.75	
^82^Se	55.52	
^99^Tc	100.00	211,000
^127^I	23.91	
^129^I	76.09	15,700,000

### Calculational method

Core characteristics were analyzed with a continuous neutron energy Monte Carlo code MVP^44^ with a JENDL-4.0^45^ neutron cross-section library. The number of neutron histories was 10,000, the number of batches skipped for accurate source distribution was 100, and the number of effective batches was 1,000. From this Monte Carlo simulation, the neutron energy spectra and the reaction rates of MAs and LLFPs in various regions of the fast breeder reactor were obtained. The typical statistical error for the k-effective was approximately 0.015% with a 1σ error. The statistical errors of the MA reaction rate in the core fuel and the reaction rate of the LLFPs in the LLFP assembly were also sufficiently low, ranging from 0.1 to 0.5%. Burnup calculations were performed with the MVP-BURN code^46^.

In the core analysis, the MVP-BURN was used to obtain changes in the k-effective and changes in Pu, MA and LLFP nuclides depending on burnup. Then, the support ratio, transmutation rate, and breeding ratio were calculated using the data on changes in Pu, MA and LLFP nuclides. The k-effective was confirmed to be 1.02 or higher at the end of the equilibrium core.

### Transmutation rate, support ratio and breeding ratio

The transmutation rate (TR) is defined as the ratio of the amount of transmuted MAs or LLFPs (per unit time) to the amount of initially loaded MAs or LLFPs:

$$ {\text{TR}} = \left( {{\text{N}}\left( 0 \right) - {\text{N}}\left( {\text{T}} \right)} \right)/{\text{N}}\left( 0 \right){\text{T}}, $$

where N(0) and T are the number of initial atoms of MAs in the core or LLFPs in the target assembly and the irradiation period, respectively.

The support ratio (SR) is defined as the ratio of the amount of transmuted MAs or LLFPs to the amount of MAs or LLFPs produced in the core fuel over the same period of time in a reactor (M),

$$ {\text{SR}} = \left( {{\text{N}}\left( 0 \right) - {\text{N}}\left( {\text{T}} \right)} \right)/{\text{M}}. $$

The breeding ratio (BR) is defined as follows:

$$ {\text{BR}} = {1} + \left( {{\text{FE}}{-}{\text{FB}}} \right)/{\text{FD}}, $$

where FD, FB and FE are the fissile material destroyed per cycle, the fissile material in the core and blankets at the beginning of the cycle and the fissile material in the core and blankets at the cycle end, respectively.
